# Patient Perception of Physician Attire in a Military Ophthalmology Clinic

**DOI:** 10.7759/cureus.12472

**Published:** 2021-01-04

**Authors:** Melanie Scheive, John Gillis, Sarah Gillis, Gary L Legault

**Affiliations:** 1 Ophthalmology, Indiana University School of Medicine, Indianapolis, USA; 2 Ophthalmology, Wilford Hall Eye Center, Lackland, USA; 3 Endocrinology, University of Texas Health Science Center at San Antonio, San Antonio, USA; 4 Ophthalmology, Brooke Army Medical Center, San Antonio, USA

**Keywords:** physician attire, professionalism, health policy, patient satisfaction, military medicine

## Abstract

Introduction

The purpose of this study is to investigate patient preferences of physician attire in an outpatient military ophthalmology clinic to determine how these preferences affect patients’ perceptions of physician competence and their overall clinical experience.

Materials and methods

This study is a prospective survey administered to patients at the ophthalmology clinic at Brooke Army Medical Center. USA. Patients who were willing to participate in a volunteer survey were included in this study. Demographic information and survey questions were utilized in this study along with words and pictures for patients to select a preference in physician attire in the clinic setting (scrubs, military uniform, or civilian professional attire) and surgical setting (surgical cap or a surgical bouffant). The survey asks patients if physician attire impacts patient confidence in physician abilities (yes or no) and if surgeon attire impacts the likelihood of the patient taking the surgeon’s advice (yes or no).

Results

The demographic distribution includes 57-77 years old participants (53%), females (61%), retirees (49%), and dependent spouses (40%). The racial distribution includes 46% - Caucasian, 20% - African American, 22% - Hispanic, 6 - % Asian, and 6% - other. Most patient appointment types were established follow-up (77%) with only 12% new and 11% walk-in. The survey results (N=308) indicate that most patients (64%) did not have a preference in physician clinical attire, while 22% preferred scrubs, 11% preferred military uniform, and 3% preferred civilian attire. Most patients (66%) did not have a preference for surgical headwear, while 27% preferred the surgical cap, and 7% preferred the surgical bouffant. Only 9% of the patients surveyed indicated that physician attire impacted their confidence in their physician’s ability, and 12% reported that attire impacted the likelihood of taking advice.

Conclusions

Most patients in an outpatient military ophthalmology clinic do not have a preference for physician attire or surgical headwear when surveyed. The majority of patients did not feel physician attire impacted their perception of physician's ability or their likelihood of taking advice. When indicating a preference, patients tended to prefer scrubs to outpatient civilian attire or military attire and trended towards preferring surgical cap over surgical bouffant for headwear.

## Introduction

Since the time of Hippocrates around 400 B.C., the physician-patient relationship has been considered sacred, requiring trust and professionalism for its establishment. Physician attire is linked to professionalism as well as patient satisfaction, confidence, and trust [1]. As a measurement of the quality of patient care in the evolving healthcare system, patient satisfaction is of considerable interest to the medical community. Patient satisfaction from clinical experience has the potential to be improved through careful consideration of patient preferences in physician attire [2-8]. The purpose of this study is to determine patient preferences in physician attire and the impact this has on patient perceptions of medical competence within a military ophthalmology clinic.

A prior meta-analysis and systemic review has indicated that patient preferences in physician attire vary in opinion and their impact on satisfaction [5]. The attribution of these discrepancies has been linked to individual study characteristics such as patient age, study location, and specialty [5, 6]. Although there have been two studies within a military context [9, 10] and two studies in civilian ophthalmology [8, 11], the medical literature does not include any studies at a robust multi-sub-specialty ophthalmology clinic in the military. Additionally, no medical literature exists on patient preferences for surgical headwear, including surgical cap and surgical bouffant, and how this influences the likelihood they will follow physician advice. In response to 2015 recommendations from the Association for peri-Operative Registered Nurses (AORN), many departments of surgery have implemented stricter surgical headwear policies in an attempt to reduce surgical site infections (SSIs), resulting in limited surgeon headwear options. A survey of 317 surgeons in all sub-specialties indicated that 70% of respondents reported a ban on cloth surgical scrub caps, 57 % reported a ban on home laundered scrubs, and 37% reported that surgical bouffant hats be exclusively worn [12]. However, a nine-month study post-implementation of these stricter policies did not show a reduction in SSIs [13]. Since surgical headwear selection does not affect patient safety based on SSI rates, patient preferences in surgical headwear are worth studying to potentially improve patient experience, and the patient-doctor relationship. This study will add to the growing body of medical literature regarding the importance of physician attire and its impact on patient care in a large military ophthalmology clinic.

## Materials and methods

A prospective survey in a large military ophthalmology clinic was completed over the course of two months from March 2018 through April 2018. The study received approval from the Internal Review Board. The survey was designed to assess patient preferences for physician attire in the clinic, including military uniform, professional civilian attire, or scrubs, as well as for physician headwear in surgery, including a surgical cap or surgical bouffant. Additionally, the survey was created to determine the impact physician attire has on patients’ perceived confidence in physician abilities and their willingness to take physician advice.

The inclusion criteria included all adult patients (>18 years old) with an appointment at the ophthalmology clinic at the Brooke Army Medical Center. Patients who declined to take the survey or had previously taken the survey were excluded. The front desk clerks distributed the anonymous survey to adult patients who had appointments at the ophthalmology clinic.

**Figure 1:**
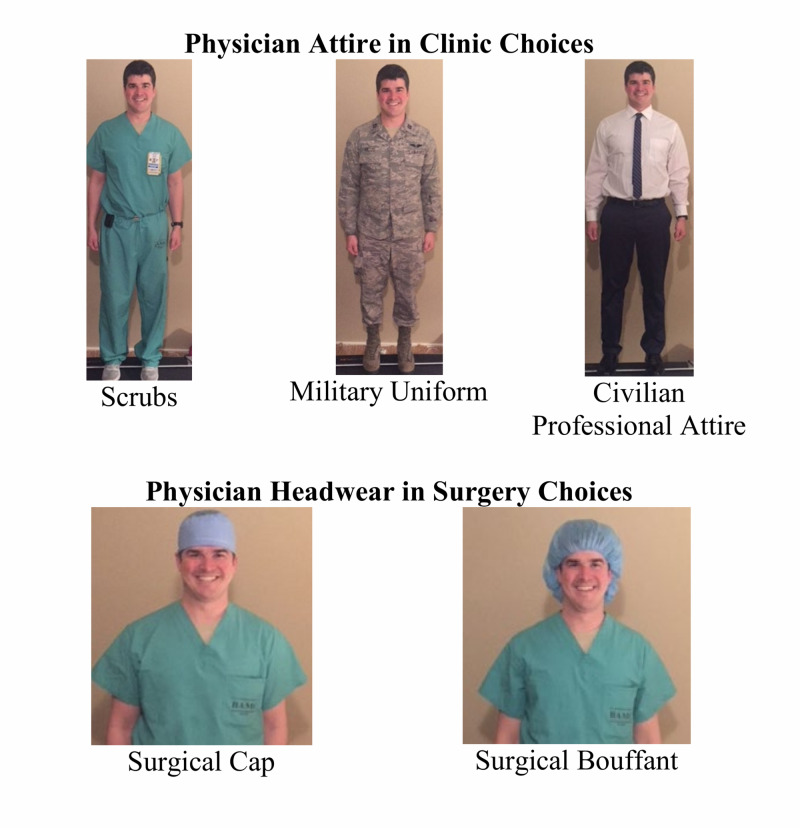
Physician attire/headwear choices on survey Images and words are reflective verbatim of those shown on the survey.

The survey included multiple-choice demographic questions to determine patient age, gender, race, beneficiary status, prior eye surgery, number of eye visits in the past year, and appointment type. The images of the physician's attire included in the survey are shown in Figure 1. The first survey question asks: “Which attire do you prefer your physician to wear?” and allows respondents to select choices of text paired with images, including the options of scrubs, military uniform, civilian professional attire, and “doesn’t matter.” The second question states: “Does your doctor’s clothes affect your confidence in their ability?” with the choices of yes, no, or “doesn’t matter.” The third question asks: “In the surgical setting, which head attire do you prefer your surgeon to wear?” and has respondents select between the options of a surgical cap, surgical bouffant, and “doesn’t matter” which pair text with images of the headwear on a surgeon. The final question states: “Does your surgeon’s attire impact the likelihood that you will take their advice?” with the answer options of yes, no, and “doesn’t matter.”

Upon completion of survey distribution, the results underwent analysis using a Chi-squared test to determine if there was a statistically significant difference in patient preferences in physician attire as well as perceptions in physician confidence and willingness to take physician advice based on demographic characteristics. Sub-group analysis was then completed on patients who indicated a preference in physician or surgical attire to evaluate any trends in opinions within this patient population. For the purposes of the study, a p-value of less than 0.05 was statistically significant.

## Results

The survey was distributed to 308 patients at the military ophthalmology clinic over the course of two months. The demographic breakdown of the study is shown in Table 1. The age distribution of study participants was 53% in the 54-77 age group, 21% in the 72-90 age group, 17% in the 38-53 age group, and 9% in the 18-37 age group. The study participant breakdown by gender was majority female (61%) and only 39% male. The beneficiary status of participants mostly consisted of retirees (48%) and dependents (40%), with only 8% identifying as active duty and 4% identifying as a civilian. The racial demographics of the study population included 46% - Caucasian, 20% - African American, 22% - Hispanic, 6% - Asian, and 6% - other. A small majority of patients (53%) did not have previous eye surgery. Most patients surveyed indicated the appointment type of established follow-up (77%), with only 12% for new and 11% for walk-in.

**Table 1:** Characteristics of study respondents All patients (N=308) responded to all the demographic questions. All options listed are worded as shown on the survey.

Characteristic	N (%)
Age	
18-37 years	28 (9.1%)
38-53 years	52 (16.9%)
54-72 years	163 (52.9%)
73-89 years	65 (21.1%)
Gender	
Female	188 (61.0%)
Male	120 (39.0%)
Race	
Caucasian	143 (46.4%)
African-American	61 (19.8%)
Hispanic	68 (22.1%)
Asian	20 (6.5%)
Other	16 (5.2%)
Beneficiary status	
Active duty	24 (7.8%)
Retiree	149 (48.4%)
Dependent	124 (40.3%)
Civilian	11 (3.6%)
Previous eye surgery	
Yes	144 (46.8%)
No	164 (53.2%)
Number of eye visits in last year	
1	124 (40.3%)
2-5	141 (45.8%)
>5	43 (14.0%)
Appointment type	
Follow-up	238 (77.3%)
New	37 (12.0%)
Walk-in	32 (10.4%)

**Figure 2:**
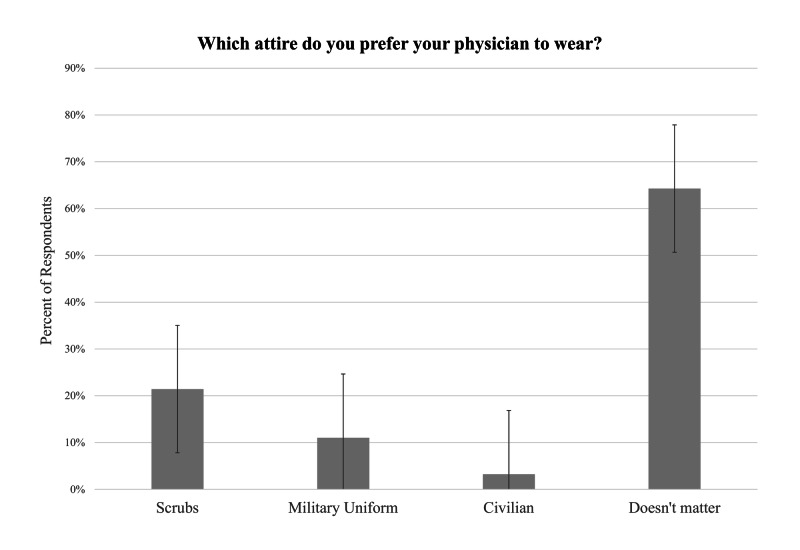
Patient preferences in clinical physician attire Standard error bars are shown for each physician attire answer choice.

**Figure 3:**
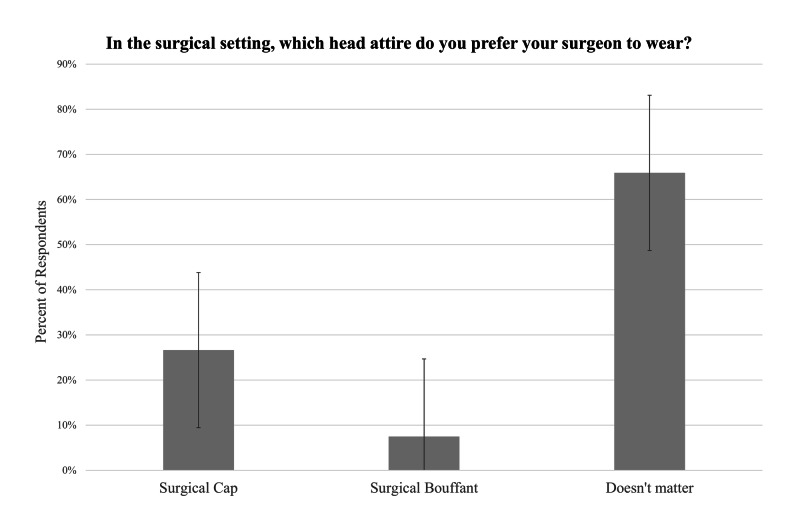
Patient preferences in surgical physician headwear Standard error bars are shown for each physician attire answer choice.

Most survey respondents did not express a preference in physician attire (64%). Out of the patients who indicated a preference in physician attire, most preferred scrubs (60%) and only 31% preferred military uniform, and 9% preferred civilian professional attire. Likewise, most patients did not report a preference in surgical headwear (66%). The surgical cap (78%) was the headwear of preference over the surgical bouffant (22%) in patients who indicated a preference. These results are illustrated with error bars of standard error in Figures 2-3. Among patients who indicated a physician attire preference, follow-up patients were more likely to prefer surgical cap compared to new or walk-in patients (p=0.004). There were no other statistically significant differences in preference between the demographic categories such as race and gender.

Out of the 308 patients surveyed, only 9% felt that physician attire influenced their confidence in the physician’s abilities, while only 12% expressed that physician headwear impacted the likelihood that they would take the physician’s advice. No statistically significant differences in responses were indicated based on the analysis of sub-groups based on patient demographics.

## Discussion

The results of the study indicate that most patients surveyed at the military ophthalmology clinic do not indicate a preference for physician attire. Among patients with a preference for attire, 60% prefer scrubs over all other attire in the military procedural outpatient clinic context. The military uniform was preferred over civilian professional attire. Historically, surgical attire is not commonly worn in the outpatient setting within surgical programs for perceived professionalism reasons [10]. However, the results of this study indicate that surgical attire is preferred by patients over all other attire in the military procedural outpatient clinic context. Additionally, the choice of physician attire influenced the confidence of 9% of patients in the abilities of their physician.

The physician attire preferences expressed by patients in this study are consistent with the results of a systematic review of patient preferences in physician attire, which indicated that four of the seven studies on procedural-based specialties like ophthalmology either showed no patient attire preference or preferred scrubs [5]. In the similarly procedure-based specialty of obstetrics and gynecology (OB/GYN), two military clinics at the Portsmouth Naval Hospital [9] and San Diego Naval Medical Center [14] similarly showed that most patients do not have a preference in physician attire but prefer scrubs when they have a preference. In the only other U.S. military setting studied, a general surgery clinic at the William Beaumont Army Medical Center, these results differed from their finding of patient preference for scrubs [10]. In the military context internationally, a study on patient attire preferences in a primary care clinic for military personnel in Saudi Arabia found the patient preference for physicians to wear the white coat, unlike this study, which may be the result of differences in the clinic and cultural contexts [15]. These results differ from most ophthalmology clinics which have been studied, including two academic and two private practices, that show a patient preference for a white coat with professional attire [8], but they are consistent with the civilian ophthalmology sub-specialty clinic, which similarly indicated that most (71%) did not have a preference or preferred scrubs and other casual attire [11]. Compared to the civilian sector, the military context in ophthalmology may reflect different patient preferences since the attire also includes the military uniform, and the population demographics uniquely include a combination of active duty personnel, military veterans, and military dependents.

The study results showed that most patients similarly did not indicate a preference in physician headwear in the surgical context. Most patients with an opinion indicated a preference toward the surgical cap more than the surgical bouffant. Twelve percent of patients surveyed indicated that physician headwear influences patient willingness to take the advice of the physician. This patient preference differs from the recommendations of AORN. There is no compelling safety evidence that indicates stricter headwear policies involving the surgical bouffant reduce SSIs [13].

The limitations of the study include that it may not be representative of all populations who attend the military ophthalmology clinic. For example, patients who have significant vision impairment may not have been able to view the images that correspond to the physician's attire and headwear options, which could impact the accuracy of their preferences. The images that depict physician attire did not encompass multiple demographics, including gender, age, and race. Patient response options were also limited to include multiple-choice options, which may not reflect the full complexity of patient opinions. Finally, the results of the study may not be applicable to civilian or other military clinic contexts because the patient demographics may be different.

Although limitations in the study exist, this study enhances our understanding of patient preferences for physician attire within a large procedural clinic context in the U.S. military. This study is the first to assess patient predilections of surgical headwear, which could potentially influence the patient-doctor relationship. The simplicity of the survey structure, along with the consistency of images of physician attire by including the same pose and facial expression for all options positively impacts the internal validity of the survey.

## Conclusions

The majority of patients in an outpatient military ophthalmology clinic do not have a preference for physician attire or surgical headwear when surveyed. Although most of our study population did not indicate a preference for physician attire, most with preferences indicated a preference of scrubs and surgical cap. A minority of patients surveyed felt that physician attire and surgical headwear options affected their confidence in their physician and likelihood of taking their advice. This study adds to the growing body of evidence that attire and surgical headwear preferences may contribute to the patient’s overall clinical experience and point to future directions of research to enhance and optimize their care.
